# Phthalate exposure and blood pressure in U.S. children aged 8–17 years (NHANES 2013–2018)

**DOI:** 10.1186/s40001-024-01785-9

**Published:** 2024-03-25

**Authors:** Tan Cheng, Chengcheng Lou, Xiaoping Jing, Sirui Ding, Haifa Hong, Guodong Ding, Li Shen

**Affiliations:** 1 Department of Cardiothoracic Surgery, Shanghai Children’s Hospital, Shanghai Jiao Tong University School of Medicine, Shanghai, China; 2 Department of Anesthesiology, Shanghai East Hospital, Tongji University, Shanghai, China; 3 Department of Traditional Chinese Medicine, Shanghai Children’s Hospital, Shanghai Jiao Tong University, School of Medicine, Shanghai, China; 4 Department of Pediatric Respiratory Medicine, Xinhua Hospital, Shanghai Jiao Tong University School of Medicine, Shanghai, 200092 China

**Keywords:** Phthalate metabolites, Blood pressure, Children, Hypertension, NHANES

## Abstract

**Background:**

Current evidence from epidemiologic studies suggested that phthalate metabolites might be associated with blood pressure (BP) changes. However, the special relationship between phthalate metabolites and BP changes in children has not been clearly elucidated in existing researches.

**Objectives:**

We investigated the links between phthalate metabolites and various BP parameters, including systolic/diastolic BP, mean arterial pressure (MAP), and the presence of hypertension.

**Methods:**

The population sample consisted of 1036 children aged 8 to 17 years from the 2013–2018 NHANES in the United States. High performance liquid chromatography-electrospray ionization-tandem mass spectrometry was used to measure urinary concentrations of 19 phthalate metabolites. Systolic/diastolic BP were derived from the average of three valid measurements, and MAP was calculated as (systolic BP + 2 × diastolic BP)/3. Hypertension was defined as mean systolic BP and/or diastolic BP that was ≥ 95th percentile for gender, age, and height reference. Linear regression, logistic regression, and weighted quantile sum (WQS) regression models were employed to assess the associations between phthalate exposure and systolic/diastolic BP, MAP, and hypertension.

**Results:**

Ten of 19 phthalate metabolites including MCNP, MCOP, MECPP, MBP, MCPP, MEP, MEHHP, MiBP, MEOHP, and MBzP had detection frequencies > 85% with samples more than 1000. MCNP, MCOP, MECPP, MBP, MCPP, MEHHP, MiBP, MEOHP, and MBzP were generally negatively associated with systolic/diastolic BP and MAP, but not protective factors for hypertension. These associations were not modified by age (8–12 and 13–17 years) or sex (boys and girls). The above-mentioned associations were further confirmed by the application of the WQS analysis, and MCOP was identified as the chemical with the highest weight.

**Conclusion:**

Phthalate metabolites were associated with modest reductions in systolic/diastolic BP, and MAP in children, while appeared not protective factors for hypertension. Given the inconsistent results among existing studies, our findings should be confirmed by other cohort studies.

**Supplementary Information:**

The online version contains supplementary material available at 10.1186/s40001-024-01785-9.

## Introduction

Phthalates are a group of synthetic chemicals manufactured at high volume and utilized in a diverse selection of industrial, consumer, medical apparatus and personal grooming commodities [1, 2]. Due to non-covalent bonding between phthalates and polymer matrices, they can easily migrate from the products into the surrounding environment [3]. Thus, phthalate metabolites can permeate human body through dermal absorption, inhalation, or ingestion [4, 5]. Once phthalates enter the human body, metabolites of phthalates could be ubiquitously detected and measured in different biological samples, including urine, blood and sweat [6–8]. The production of phthalate globally rose from 2.7 to an estimated 6 million tons per year between 2007 and 2017 [9]. In recent decades, there has been growing concern about the impact of phthalates on human health due to their widespread use and exposure. Several previous studies have reported detectable levels of phthalate metabolites in human urine, including children and pregnant women [10, 11]. Researches have shown that long-term exposure to phthalates is associated with a rage of health effects, including endocrine disruption and neurotoxicity, carcinogenicity, reproductive and developmental toxicity [12–14].

In recent years, the prevalence of hypertension in children has been on the rise [15]. A meta-analysis reported a worldwide increment in the prevalence of hypertension from 75 to 79% in children aged 6 to 19 years, from 2000 to 2015 [16]. The factors influencing BP changes are complex, including emotion, living and dietary habits, sleep duration and even environmental exposure [17–19]. Among these causes, chemical toxicants have been proposed frequently as important risk factors that could affect childhood BP. Several epidemiologic studies have examined the relationships between phthalate metabolites and BP among general adult population, pregnant women, and children, while produced inconsistent results, with some reporting negative associations [20], some showing positive associations [21], and others revealing no associations [22]. For example, a prospective birth cohort study in Wuhan, China between 2013 and 2015 found that the exposure to phthalates was positively related to BP in 636 pregnant women [23]. However, the Prospective Investigation of the Vasculature in Uppsala Seniors (PIVUS) study showed that MEP concentration had a negative relation to diastolic BP but not to systolic BP in Caucasians aged 70 years [24]. In addition, the results from different cohort studies appeared contradictory in children. A cross-sectional study in Wuhan recruiting 276 children between aged 6 and 8 years suggest that there was an association between MEP and MEOHP concentrations and systolic BP among boys, however, no such associations were found among girls within the same age group. Additionally, MEHHP concentration exhibited a positive correlation with diastolic BP among boys, but not among girls [25]. On the contrary, a study recruiting 242 participants aged 6 to18 years in Iran showed that MBP concentration was positively related to increased BP [26].

Therefore, given children are exposed to phthalates more extensively at higher levels than adults [27], and inconsistent findings between exposure metabolites and childhood BP, the objective of this study was to assess the relationship between urinary concentrations of phthalate metabolites with BP in children aged 8–17 years, utilizing data from the U.S. NHANES study conducted between 2013 and 2018.

## Methods

### Study population

In this research, we selected participants from the NHANES database, which includes a combination of demographic data, dietary data, examination data and laboratory data to evaluate the healthy status of population in U.S. This study received approval from the Research Ethics Review Board at the National Centre for Health Statistics, and each participant provided informed consent. More detailed descriptions about the NHANES study are available on the Web site (http://www.cdc.gov/nchs/nhanes.htm).

**Fig. 1:**
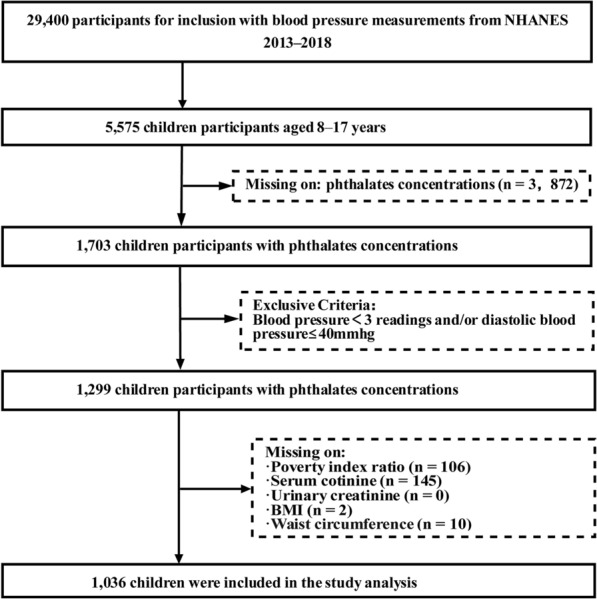
Participant’s selection flowchart

We summarized the study population from the three successive cycles of NHANES (2013**–**2018), including data from a total of 29,400 participants. A total of 5575 participants aged of 8 to 17 years were included in our study. Of these, we excluded 3872 children with missing data on phthalate exposure. Additionally, we excluded participants who had missing values for systolic and diastolic BP measures, and/or diastolic blood pressure ≤ 40 mmhg (*n* = 404), BMI (*n* = 2), waist (*n* = 10), family income-to-poverty ratio (*n* = 106), serum cotinine (*n* = 145), and urinary creatinine (*n* = 0). Therefore, 1036 children constituted the final study population, as shown in Figure 1.

### Exposure information

One-third of the study participants aged 6 years or older had random urine samples of phthalate metabolites taken for quantification. The gathered specimens were frozen at – 20 ℃ and dispatched for evaluation to the National Center for Environmental Health of the CDC. Urine samples were analyses by high-performance (HPLC-ESI-MS/MS) for liquid chromatography-electrospray ionization-tandem mass spectrometry (HPLC-ESI-MS/MS) to quantify phthalate metabolites.

Urinary concentrations of nineteen phthalate metabolites were measured, and the limit of detection (LOD) for the phthalate metabolites was 0.2 ng/mL for mono-(carboxyisononyl) phthalate (MCNP), 0.3 ng/mL for mono-(carboxyisoctyl) phthalate (MCOP), 0.4 ng/mL for mono-2-ethyl-5-carboxypentyl phthalate (MECPP), 0.4 ng/mL mono-butyl phthalate (MBP), 0.4 ng/mL for mono-(3-carboxypropyl) phthalate (MCPP), 1.2 ng/mL for mono-ethyl phthalate (MEP), 0.4 ng/mL for mono-(2-ethyl-5-hydroxyhexyl) phthalate (MEHHP), 0.8 ng/mL for mono-isobutyl phthalate (MiBP), 0.2 ng/mL for mono-(2-ethyl-5-oxohexyl) phthalate (MEOHP), 0.3 ng/mL for mono-benzyl phthalate (MBzP), 0.4 ng/mL for cyclohexane 1,2-dicarboxylic acid monohydroxy isononyl ester(MHNCH), 0.8 ng/mL for mono-(2-ethylhexyl) phthalate (MEHP), 0.9 ng/mL for monoisononyl phthalate (MNP), 0.5 ng/mL for Cyclohexane-1,2-dicarboxylic acid-mono(carboxyoctyl) ester phthalate (MCOCH), mono-2-ethyl-5-carboxypentyl terephthalate (MECPTP), 0.4 ng/mL for mono-2-hydroxy-iso-butyl phthalate (MHIBP), 0.4 ng/mL for mono-3-hydroxy-n-butyl phthalate (MHBP), 0.4 ng/mL for mono-2-ethyl-5-hydroxyhexylterephthalate (MEHHTP) and 0.4 ng/mL for monooxoisononyl phthalate (MONP). The LOD divided by the square root of two was used to replace the concentrations below the limit of detection (LOD). In this study, regression analyses were restricted to the ten phthalate metabolites with high detection frequency (> 80%) and enough sample size (> 1000), including MCNP, MCOP, MECPP, MBP, MCPP, MEP, MEHHP, MiBP, MEOHP, and MBzP.

The comprehensive details regarding detection techniques and quality control procedures could be found online at http://www.cdc.gov/nchs/nhanes.htm. Urine concentrations of creatinine were measured by the modifiedJaffe colorimetric method using automated Beckman analyzers (Beckman Instruments, Inc., Brea, CA). The urinary concentrations of pathalate metabolites were adjusted using creatinine levels in the spot urine samples for correcting variation in urine dilution.

### Demographic covariates

These demographic covariates were collected through the self-reported questionnaires, physical examinations, and laboratory measurements. Age, gender, race/ethnicity, and poverty status were included in the self-reported questionnaires. Poverty status was determined by calculating the PIR, which divided the family income by the poverty thresholds of a particular survey year. Ethnicity was categorized as “non-Hispanic white”, “non-Hispanic black”, “Mexican American,” and “Other”. Participants were categorized as underweight (BMI < 5th percentile), normal weight (BMI 5th to < 85th percentile), overweight (BMI 85th to < 95th percentile), or obese (BMI ≥ 95th percentile). The body measures about waist circumference were measured by trained health technicians in NHANES. Serum cotinine served as a biomarker for smoking status, with levels classified as below the limit of detection (LOD), less than 1 ng/mL, and 1 ng/mL or higher.

### Outcome assessment

All NHANES participants aged 8 years or older were eligible to undergo BP measurements at the mobile examination stations. Three consecutive BP measurements were made after the participant sat still for 5 min. And if a measurement was interrupted or incomplete, a fourth attempt should be made to re-measure. Mean systolic BP and diastolic BP were computed using the first three readings. The mean arterial pressure (MAP) was calculated as follows: MAP = (systolic BP + (2 × diastolic BP))/3. Based on the Fourth Report of National High Blood Pressure Education Program Working Group on hypertension in Children and Adolescents, hypertension is characterized as mean systolic BP and/or diastolic BP that is equal to or greater than the 95th percentile for gender, age, and height reference [28].

### Statistical analysis

Summary statistics presented the demographics, urinary levels of phthalate metabolites, and measurements of systolic/diastolic BP and MAP of the research sample. Means and standard deviations (SDs) or medians and interquartile ranges or frequency distributions were used to present descriptive data for population characteristics. Demographic characteristics were compared using the Mann–Whitney U test for continuous variables and chi-squared test for categorical variables between the phthalate baseline data set (*n* = 5575) and the study cohort (*n* = 1036).

Linear regression was performed to investigate the associations between urinary concentrations of phthalate metabolites and systolic/diastolic BP and MAP in children. The continuous variables of phthalate metabolites, systolic/diastolic BP, and MAP had non-normal distributions, thus, we did log_10_-transformation in the analysis. Logistic regression was performed to investigate the correlation of phthalate metabolites with risk of high BP (Yes/no). Stratified subgroup analyses were further conducted to examine whether age (8–12 and 13–17 years) and sex (boys and girls) differences modified these associations. On the basis of the meaningful outcomes of linear regression analysis, weighted quantile sum (WQS) models were further employed to assess the cumulative effect of phthalate metabolites on BP in children. In brief, WQS regression could estimate the contributing effects of each individual exposure of the combined association [29, 30].

In the present study, we created WQS index and estimated the weighting of each exposure for its contribution to the overall mixing effect. Based on no precedent guidance on the direction of the associations with BP for the individual chemicals, we performed two types of WQS regression tests: one test was based on the assumption that all index components had a positive correlation with BP, while the other test was based on the assumption that all index components had a negative correlation with BP.

SPSS (v25.0; SPSS Inc., Chicago, IL) and R (v4.0.3) were used for statistical analysis. Statistical significance was defined as a two-sided *p*-value < 0.05.

## Results

The socio-demographic characteristics of 1036 children aged 8–17 years are presented in Table 1. The average (SD) age and waist were 12.61 (2.89) years and 77.85 (15.53) cm, respectively. The majority (80.7%) of the participants were normal weight, approximately half (49.0%) were boys, and nearly one-thirds (29.5%) were non–Hispanic white. The study population was generally representative of the larger NHANES participant group, as there were no differences in demographic characteristics between the 1299 children in the phthalate data set and the study population (Additional file 1: Table S1).

**Table 1:** Characteristics stratified by sex in children aged 8–17 years: NHANES 2013–2018 (n = 1,036)

	Total	8–12 years		13–17 years	
	N (%) or mean ± SD	Boys	Girls	Boys	Girls
		*n* = 243	*n* = 263	*n* = 265	*n* = 265
Characteristics					
Age (years)					
	12.61 (2.89)	10.02 (1.38)	9.99 (1.32)	15.12 (1.38)	15.07 (1.42)
BMI (kg/m^2^)^a^					
Underweight	42 (4.1)	19 (7.8)	20 (7.6)	0 (0.0)	13 (4.9)
Normal	836 (80.7)	208 (85.6)	224 (85.2)	204 (77.0)	213 (58.4)
Overweight	107 (10.3)	12 (4.9)	13 (4.9)	37 (14.0)	26 (9.8)
Obese	51 (4.9)	4 (1.7)	6 (2.3)	24 (9.0)	13 (4.9)
Waist circumference (cm)					
	77.85 (15.53)	71.97 (13.69)	72.14 (12.77)	84.78(17.31)	81.96 (13.53)
Race					
Non–Hispanic White	306 (29.5)	78 (32.1)	67 (25.5)	92 (34.7)	69 (26.0)
Non–Hispanic Black	220 (21.2)	54 (22.2)	57 (21.7)	54 (20.4)	55 (20.8)
Mexican American	245 (23.6)	56 (3.1)	69 (26.2)	57 (21.5)	63 (23.8)
Other Race	265 (25.6)	55 (22.6)	70 (26.6)	62 (23.4)	78 (29.5)
Poverty index ratio (PIR)					
Below poverty level (< 1.85)	606 (58.5)	133 (54.7)	167 (63.5)	145 (54.9)	161 (60.8)
Above poverty level (≥ 1.85)	430 (41.5)	110 (45.3)	96 (36.5)	119 (45.1)	104 (39.2)
Serum cotinine level (ng/mL)					
< LOD	394 (38.0)	90 (37.0)	105 (41.5)	79 (29.8)	110 (41.5)
≥ LOD	642 (62.0)	153 (63.0)	148 (58.5)	186 (70.2)	155 (58.5)
NHANES cycles					
2013–2014	363 (35.0)	74 (30.5)	97 (36.9)	96 (36.2)	96 (36.2)
2015–2016	394 (38.0)	111 (45.7)	98 (37.3)	96 (36.2)	89 (33.6)
2017–2018	279 (27.0)	58 (23.8)	68 (25.8)	73 (27.6)	80 (30.2)

^a^ NHANES classified participants as underweight (BMI < 5th percentile), normal weight (BMI 5th to < 85th percentile), overweight (BMI 85th to < 95th percentile), or obese (BMI ≥ 95th percentile) according to the U.S. CDC gender-specific growth charts based on age in months and years

Urinary concentrations of 19 phthalate metabolites, both unadjusted (μg/L) and adjusted for creatinine (μg/g), are shown in Table 2. Among these, the 10 metabolites including MCNP, MCOP, MECPP, MBP, MCPP, MEP, MEHHP, MiBP, MEOHP, and MBzP had detection frequencies > 85% with samples more than 1000.

**Table 2:** Distributions of urinary concentrations of phthalate metabolites in children aged 8–17 years: NHANES 2013–2018 (*n* = 1,036)

Metabolites	Detection rates *N* (%)	LODs	Not adjusted for creatinine (μg/L)				Creatinine adjusted (μg/g)			
			Selected percentiles				Percentiles of distribution			
			25th	50th	75th	95th	25th	50th	75th	95th
MCNP	1024/1036 (98.84)	0.20	1.20	2.30	4.20	12.53	1.16	1.95	3.46	9.27
MCOP	1034/1036 (99.80)	0.30	5.10	10.40	25.98	149.49	4.61	9.43	22.59	116.25
MECPP	1036/1036 (100.0)	0.40	6.40	12.65	22.20	51.81	6.28	10.57	17.74	43.01
MBP	1031/1036 (99.52)	0.40	6.90	13.50	24.38	54.52	7.08	11.41	18.72	38.46
MCPP	912/1036 (88.03)	0.40	0.80	1.60	3.10	11.52	0.74	1.38	2.68	9.13
MEP	1033/1036 (99.71)	1.20	13.23	29.10	70.78	368.96	13.76	24.39	55.01	231.03
MEHHP	1032/1036 (99.61)	0.40	3.70	7.30	13.90	32.12	3.66	6.14	10.97	25.72
MiBP	1026/1036 (99.03)	0.80	5.30	10.70	19.80	51.36	5.66	9.28	14.87	35.88
MEOHP	1033/1036 (99.71)	0.20	2.53	5.00	9.30	21.50	2.52	4.22	7.26	17.25
MBzP	1029/1036 (99.32)	0.30	3.30	7.15	16.18	50.29	3.27	5.91	12.88	38.72
MCOCH^a^	408/673 (60.62)	0.50	< LOD	0.60	1.00	4.53	< LOD	0.58	1.10	3.62
MHNCH	689/1036 (66.51)	0.40	< LOD	0.60	1.40	5.03	< LOD	0.63	1.32	4.34
MEHP	668/1036 (64.48)	0.80	< LOD	1.10	2.10	6.10	< LOD	1.20	2.13	5.43
MNP	341/1036 (32.91)	0.90	< LOD	< LOD	1.20	8.70	< LOD	< LOD	1.52	6.88
MECPTP^b^	279/279 (100.00)	0.20	17.50	40.40	102.50	499.60	15.91	34.00	75.00	471.90
MHIBP^c^	655/673 (97.33)	0.40	1.80	3.60	6.60	15.49	1.93	3.08	5.10	11.96
MHBP^d^	569/673 (84.55)	0.40	0.60	1.20	2.30	4.80	0.63	1.10	1.80	3.87
MEHHTP^e^	276/279 (98.92)	0.40	3.80	8.70	19.60	95.40	3.62	6.47	14.68	79.12
MONP^f^	253/279 (90.68)	0.40	0.90	1.70	3.40	12.90	0.81	1.40	2.60	9.91

^a^MCOCH is available from two cycles including 2013–2014 and 2015–2016

^b^MECPTP is available from one cycle including 2017–2018

^c^MHIBP is available from two cycles including 2015–2016 and 2017–2018

^d^MHBP is available from two cycles including 2015–2016 and 2017–2018

^e^MHHT is available from one cycle including 2017–2018

^f^MONP is available from one cycle including 2017–2018

Distributions of blood pressure indices including systolic/diastolic BP, and mean arterial pressure (MAP) by age category (8–12 and 13–17 years) and sex category (boys and girls) of this study population are shown in Table 3. The older children and the boys were more likely to have higher systolic/diastolic BP, and MAP.

**Table 3:** Blood pressure measures in children aged 8–17 years: NHANES 2013– 2018 (n = 1,036)

Blood Pressure (mmHg)	Mean ± SD	Selected percentiles			
		25th	50th	75th	95th
Total					
Systolic BP	105.86 (9.45)	99.33	105.33	112.00	122.67
Diastolic BP	60.22 (8.60)	53.33	60.00	66.67	74.10
Mean arterial pressure	75.43 (7.20)	70.00	75.33	80.22	87.56
Age					
8–12 years					
Systolic BP	102.85 (8.63)	97.33	102.00	108.67	117.33
Diastolic BP	58.35 (8.26)	52.00	58.00	64.00	73.10
Mean arterial pressure	73.19 (6.17)	68.39	72.89	77.56	85.11
13–17 years					
Systolic BP	108.74 (9.30)	102.67	108.00	114.67	124.00
Diastolic BP	62.00 (8.54)	56.00	62.00	68.00	75.33
Mean arterial pressure	77.58 (6.94)	70.00	76.00	84.00	96.00
Sex					
Boys					
Systolic BP	107.71 (9.83)	100.67	107.33	113.33	124.00
Diastolic BP	60.17 (8.85)	53.33	60.00	66.67	74.67
Mean arterial pressure	76.02 (7.39)	70.44	75.56	80.89	88.22
Girls					
Systolic BP	104.09 (8.71)	98.67	104.00	109.33	118.37
Diastolic BP	60.27 (8.36)	53.50	60.67	66.50	74.00
Mean arterial pressure	74.87 (6.96)	69.78	75.11	79.56	86.67

Relationships of urinary concentrations of 10 phthalate metabolites with systolic/diastolic BP, MAP, and hypertension are presented in Table 4. Nine of the ten metabolites including MCNP, MCOP, MECPP, MBP, MCPP, MEHHP, MiBP, MEOHP, and MBzP were generally correlated with modest reduction in systolic/diastolic BP and MAP, but not associated with decreased risks of hypertension. No associations were found between MEP and the four BP outcomes. Stratified subgroup analyses were performed to examine whether age (children aged 7–12 years and adolescents aged 13–17 years) or sex (boys and girls) difference modified these associations, while the results in sex- or age-stratified analysis showed a similar trend to the overall population (Additional file 1: Tables S2-S3).

**Table 4:** Association between urinary concentrations of phthalate exposure and systolic/diastolic BP, mean arterial pressure (MAP), and hypertension in children in the NHANES 2013–2018 (n = 1,036)

	Systolic BP		Diastolic BP		Mean arterial pressure		Hypertension	
Exposures	Adjusted β (95% CI)^a^						Adjusted OR (95% CI)^a^	
MCNP	− 0.011 (− 0.017, − 0.005)	< 0.001	− 0.015 (− 0.025, − 0.005)	0.003	− 0.123 (− 0.007, − 020)	< 0.001	1.004 (0.978, 1.031)	0.761
MCOP	− 0.011 (− 0.015, − 0.007)	< 0.001	− 0.013 (− 0.020, − 0.006)	0.001	− 0.153 (− 0.017, − 0.007)	< 0.001	0.998 (0.995, 1.002)	0.353
MECPP	− 0.020 (− 0.026, − 0.013)	< 0.001	− 0.028 (− 0.038, − 0.017)	< 0.001	− 0.208 (− 0.031, − 0.017)	< 0.001	0.999 (0.995, 1.004)	0.801
MBP	− 0.019 (− 0.026, − 0.012)	< 0.001	− 0.018 (− 0.029, − 0.006)	0.003	− 0.144 (− 0.026, − 0.011)	< 0.001	0.996 (0.986, 1.006)	0.443
MCPP	− 0.016 (− 0.021, − 0.010)	< 0.001	− 0.017 (− 0.026, − 0.008)	< 0.001	− 0.172 (− 0.022, − 0.011)	< 0.001	0.998 (0.964, 1.003)	0.914
MEP	− 0.001 (− 0.006,0.004)	0.793	− 0.005 (− 0.013, 0.003)	0.220	− 0.035(− 0.008, 0.002)	0.265	1.000 (0.999, 1.001)	0.862
MEHHP	− 0.014 (− 0.021, − 0.080)	< 0.001	− 0.024 (− 0.034, − 0.014)	< 0.001	− 0.178 (− 0.026, − 0.013)	< 0.001	1.000 (0.995, 1.005)	0.892
MiBP	− 0.017 (− 0.024, − 0.010)	< 0.001	− 0.023 (− 0.034, − 0.012)	< 0.001	− 0.169 (− 0.028, − 0.013)	< 0.001	0.998 (0.992, 1.003)	0.419
MEOHP	− 0.018 (− 0.025, − 0.012)	< 0.001	− 0.027 (− 0.037, − 0.016)	< 0.001	− 0.198 (− 0.030, − 0.016)	< 0.001	0.999 (0.991, 1.008)	0.842
MBzP	− 0.009 (− 0.015, − 0.040)	< 0.001	− 0.014 (− 0.022, − 0.005)	0.001	− 0.127 (− 0.017, − 0.006)	< 0.001	1.004 (0.997, 1.011)	0.286

^a^Adjusted for age, sex, race, waist, BMI, serum cotinine level, and family income to poverty ratio, and NHANES cycles

**Fig. 2:**
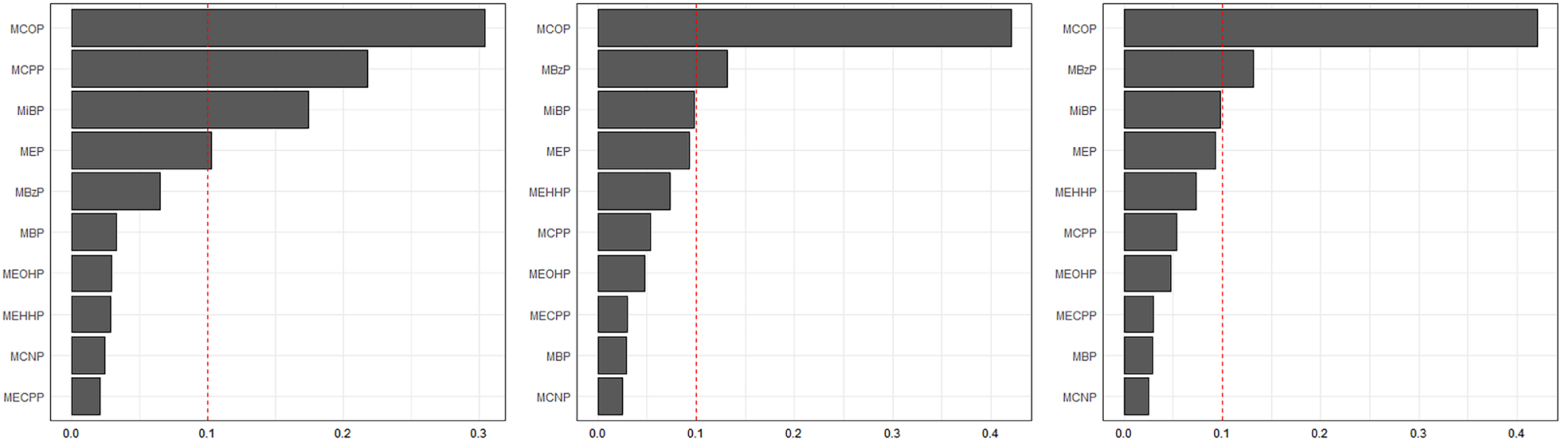
Weights of all measured phthalate metabolites in association with BP indices using WQS regression in total participants aged 8–17 years: NHANES 2013–2014. Adjusted for age, sex, race, waist, BMI, serum cotinine level, and family income to poverty ratio, and NHANES cycles

The aforementioned significantly negative relationships in linear regression were additionally supported by the use of WQS analysis. Table 5 shows the WQS weightings and regression index of the phthalate compounds with systolic/diastolic BP and MAP in the negative WQS model. In the negative WQS models, significantly negative associations were detected of chemical compounds with systolic BP (*p* <0.01), diastolic BP (*p* < 0.05), and MAP (*p* < 0.01) in the whole population. MCOP was identified as the chemical with the greatest weight in the negative associations (weight=0.30 for S BP, 0.42 for DBP, and 0.37 for MAP) (Figure 2). The results in sex- and age-stratified analysis showed a similar trend to the overall population (Table 5, and Additional file 1: Figure S1–S2).

**Table 5:** Associations between WQS index and BP indices from the NHANES 2013–2018 (n = 1,036)

Models	Population		Systolic BP	Diastolic BP	Mean arterial pressure
			Adjusted β (95% CI)		
Negative	Total		− 0.005(− 0.008, − 0.002)**	− 0.007( − 0.013, − 0.001)*	− 0.001(− 0.009, − 0.002)**
	Sex				
		Males (*n* = 508)^b^	− 0.004(-0.009, 0.000)	− 0.010(− 0.019, − 0.001)**	− 0.007(− 0.012, − 0.002)*
		Females (*n* = 528)^b^	− 0.006(− 0.011, − 0.001)*	− 0.006(− 0.010, 0.000)	− 0.005(− 0.010, 0.000)
	Age				
		8–12 (*n* = 506)^c^	− 0.007(− 0.078, − 0.069)**	− 0.008(− 0.001, − 0.069)*	− 0.007(− 0.012, − 0.003)**
		13–17 (*n* = 530)^c^	− 0.004(− 0.008, 0.000)	− 0.012(− 0.020, − 0.003)**	− 0.008(− 0.013, − 0.022)**

^a^Adjusted for age, sex, race, waist, BMI, serum cotinine level, and family income to poverty ratio, and NHANES cycles

^b^Adjusted for age, race, waist, BMI, serum cotinine level, and family income to poverty ratio, and NHANES cycles

^c^ Adjusted for sex, race, waist, BMI, serum cotinine level, and family income to poverty ratio, and NHANES cycles

^*^P < 0.05

^**^P < 0.01

## Discussion

In the present study, we evaluated the links of phthalate exposures with BP among children aged 8–17 years based on the US data set NHANES 2007-2012. Our findings showed that phthalate metabolite concentrations were correlated with modest decreases in systolic/diastolic BP, and MAP in children, and MCOP was found as the most significant chemical. However, phthalate exposure appeared not a protective factor for hypertension.

Several previous studies investigated the potential correlations of phthalate exposures with BP among children, with inconsistent results. For example, a cross-sectional study from Wuhan, China recruiting 276 children between the ages of 6–8 years found negative relationships between MEP and MEOHP concentrations and systolic BP, while positive association between MEHHP concentrations and diastolic BP among boys. However, no associations were found among girls [25]. A prospective cohort of 500 mother-child pairs at the prefecture of Heraklion, Greece reported that consistently negative correlations of phthalate metabolite concentrations at age 4 with systolic/diastolic BP overall and in boys aged 4–6 years. Prenatal exposure to MiBP in early pregnancy was negatively related to diastolic BP overall and in boys at 4–6 years, but not in systolic BP. [20]. Another birth cohort study in 391 mother-child pairs in Sabadell, Spain revealed that both high- and low-molecular phthalate metabolites were linked to reduced systolic BP at 4–7 years of age in girls but not in boys, while no significant association with diastolic BP was found [21]. In contrast, a recent cross-sectional study from Shenzhen, China, between 2016 and 2017 found some kinds of phthalates were related to increased systolic/diastolic BP, pulse pressure, and MAP in Chinese children. Another study from Isfahan, Iran recruiting 242 children and teenagers aged between 6 and 18, suggested that significant association existed between MBP and increased BP [26]. Our results partly supported the findings from the previous studies. In our study, concentrations of phthalate metabolites and blood pressure in children were found to be negatively associated, while phthalate exposures appeared not a protective factor for hypertension. Differences in exposure scenarios, sample sizes, study populations and BP assessment techniques make direct comparisons with existing studies difficult. For example, the different levels of exposure may explain the inconsistent results. Median phthalate metabolite concentrations in study of Isfahan, Iran were 30 times higher more than in our study (i.e., MBzP: 240.9 vs 7.1 μg/*g* creatinine) [26]. What’s more, the above-mentioned associations may differ according to the age and race of the children. Animal toxicology studies had shown that di-2-ethylhexyl phthalate can increase blood pressure in mice and rats, while its primary metabolite, MEHP, can significantly reduce both heart rate and blood pressure [31]. Our results were in partial agreement with results from the animal studies. In addition, daily phthalate exposure is much larger than that found in animal studies in people. Animals are exposed to phthalates at doses of up to grams per kilogram, but the highest human exposure is found through haemodialysis and total parenteral nutrition in young children. Therefore, there is still a great uncertainty about the level of human exposure to the various phthalates and phthalate mixtures that may have cumulative effects. Phthalates and their metabolites may bioaccumulate and the exact concentrations that accumulate in the body are not yet known. Urinary concentrations of phthalates or their metabolites can be as high as 305 μg/L, therefore, it is essential to possess adequate comprehension of the levels at which there is a risk of adverse effects. Based on varying sources of phthalate exposure concentrations and durations, it is crucial to investigate a wide range of concentrations and ascertain the minimum level of adverse effects that were observed.

Epidemiologic studies investigating the sex-specific responses on association of phthalate metabolites with BP in children were scarce, and reported less consistent results, with several studies suggesting more pronounced among boys (Wu et al. 2018; Vafeiadi et al. 2018), while the others indicating stronger among girls (Valvi et al. 2015). In our study, we found that the associations between phthalate exposures and BP were not modified by sex or age. We need more studies to explore the sex or age difference regarding the phthalate metabolites on BP and to further insight the underlying mechanisms.

In fact, various methods have been utilized to assess the impact of chemical substances in the environment on human health. However, the associations between phthalate exposures and BP using WQS models are relatively lacking in current studies. Generally, the regression analysis conducted on the WQS data reinforces the significant outcomes revealed by our linear regression analysis. The present study notes that the MCOP was shown to be the primary chemical contributing significantly to the total compound impact on BP. Although the precise mechanisms are currently unclear, compelling evidence has revealed that the dialkyl or alkyl/aryl side chains determine the toxicity of phthalates [32, 33]. MCOP was the monoester metabolites of di-isononyl phthalate (DINP). And the oral LD50 for DINP exceeds 10 *g*/kg, indicating lower toxicity than DEHP and rendering it a preferred substitute. As a consequence, the use of DINP has been found to be excessive. Our findings may partly be explained by this phenomenon of overuse.

The strength of this study lies in its nationally representative sample design, with information on relevant potential confounders available, which made our results both generalizable and reliable. However, there are some limitations to interpret. First, the causal relationship between concentrations of metabolites and BP cannot be unequivocally made due to the cross-sectional nature of the study. A large prospective study of phthalate is essential to explore the possible relationships. Second, we just took into account the effects of phthalate metabolites on BP, however, a wide range of environmental chemicals have an adverse effect BP were not included. Third, in NHANES, a single spot urine sample was collected for the analysis of phthalate metabolites, which may lead to increased variability issues compared to using of 24-hour urine samples. Previous studies within the NHANES cohorts have substantiated that phthalate concentrations bear correlations with a plethora of lifestyle factors and architectural attributes [34]. The inherent stability of said lifestyle and architectural determinants may culminate in a sustained exposure to chemicals with shorter biological half-lives.

## Conclusion

In conclusion, our study implies that phthalate metabolites were associated with modest reductions in systolic/diastolic BP and MAP among children aged 8–17 years, while appeared not protective factors for hypertension. Among the negative effects of plasticizers on blood pressure, MCOP contributed the most. Based on the cross-sectional design and inconsistent results, additional studies should be warranted to evaluate the correlation between phthalate metabolites and blood pressure in children.

### Supplementary Information

**Additional file 1: Figure S1.** Sex stratified association between urinary phthalate metabolites concentration and BP indices: boys (**A**) and girls (**B**). Adjusted for age sex, race, waist, BMI, NHANES cycles, serum cotinine level and family income to poverty ratio. **Figure S2.** Age stratified association between urinary phthalate metabolites concentration and BP indices: 8–12 years (**A**) and 13–17 years (**B**). Adjusted for sex, age, race, waist, BMI, NHANES cycles, serum cotinine level and family income to poverty ratio. **Table S1. **Demographic characteristics of all participants 8–17 years of age (*n* = 5575) and the study population (*n* = 1036). ^a^Proportions were compared by Pearson chi-square test. ^b^ Mean rank was compared by Mann-Whitney U Test. **Table S2. **Association between urinary concentrations of phthalate metabolites and systolic/diastolic BP, mean arterial pressure (MAP), and hypertension among boys and girls in the NHANES 2013–2018 (*n* = 1036). ^a^Adjusted for age, race, waist, BMI, serum cotinine level, and family income to poverty ratio, and NHANES cycles. **Table S3. **Association between urinary concentrations of phthalate metabolites and systolic/diastolic BP, mean arterial pressure (MAP), and hypertension in children aged 8–17 years in the NHANES 2013–2018 (*n* = 1036). ^a^Adjusted for sex, race, waist, BMI, NHANES cycles, serum cotinine level and family income to poverty ratio.

## Data Availability

The data we used and analyzed in our study are available from https://www.cdc.gov/nchs/nhanes.

## Abbreviations

MCNP: Mono(carboxyisononyl) phthalate
MCOP: Mono-carboxyoctyl phthalate
MECPP: Mono(2-ethyl-5-carboxypentyl) phthalate
MBP: Monobutyl phthalate
MCPP: Mono-(3-carboxypropyl) phthalate
MEP: Monoethyl phthalate
MEHHP: Mono-(2-ethyl-5-hydroxyhexyl) phthalate
MHNCH: Cyclohexane-1,2-dicarboxylic acid-mono (hydroxy-isononyl) ester
MEHP: Mono-(2-ethylhexyl) phthalate
MiBP: Mono-isobutyl phthalate
MNP: Monoisononyl phthalate
MEOHP: Mono-(2-ethyl-5-oxohexyl) phthalate
MBzP: Monobenzyl phthalate
MCOCH: Cyclohexane-1,2-dicarboxylic acid-mono(carboxyoctyl) ester phthalate
MECPTP: Mono-2-ethyl-5-carboxypentyl terephthalate
MHIBP: Mono-3-hydroxy-n-butyl phthalate
MEHHTP: Mono-2-ethyl-5-hydroxyhexylterephthalate
MONP: Monooxoisononyl phthalate
BP: Blood pressure
MAP: Mean arterial pressure
NHANES: National Health and Nutrition Examination Survey
WQS: Weighted quantile sum
NCHS: National Center for Health Statistics
LOD: Limit of detection
PIR: Poverty income ratio
BMI: Body mass index
95% CI: 95% Confidence interval
SD: Standard deviation
