# Supplementary material for: Phthalate exposure and blood pressure in U.S. children aged 8–17 years (NHANES 2013–2018)
Source: Eur J Med Res. 2024 Mar 25;29:192. doi: 10.1186/s40001-024-01785-9 (PMC10962091; doi:10.1186/s40001-024-01785-9)
Supplement: Supplementary file 1 — Additional file 1: Figure S1. Sex stratified association between urinary phthalate metabolites concentration and BP indices: boys (A) and girls (B). Adjusted for age sex, race, waist, BMI, NHANES cycles, serum cotinine level and family income to poverty ratio. Figure S2. Age stratified association between urinary phthalate metabolites concentration and BP indices: 8–12 years (A) and 13–17 years (B). Adjusted for sex, age, race, waist, BMI, NHANES cycles, serum cotinine level and family income to poverty ratio. Table S1. Demographic characteristics of all participants 8–17 years of age (n = 5575) and the study population (n = 1036). aProportions were compared by Pearson chi-square test. b Mean rank was compared by Mann-Whitney U Test. Table S2. Association between urinary concentrations of phthalate metabolites and systolic/diastolic BP, mean arterial pressure (MAP), and hypertension among boys and girls in the NHANES 2013–2018 (n = 1036). aAdjusted for age, race, waist, BMI, serum cotinine level, and family income to poverty ratio, and NHANES cycles. Table S3. Association between urinary concentrations of phthalate metabolites and systolic/diastolic BP, mean arterial pressure (MAP), and hypertension in children aged 8–17 years in the NHANES 2013–2018 (n = 1036). aAdjusted for sex, race, waist, BMI, NHANES cycles, serum cotinine level and family income to poverty ratio. [file 40001_2024_1785_MOESM1_ESM.zip › New folder/Table S1.docx]

| **Table S1** Demographic characteristics of all participants 8–17 years of age (n =1,299) and the study population (n= 1,036) | | | | | |
| --- | --- | --- | --- | --- | --- |
|  | Phthalates data  (n=1, 299) | | Study population  (n=1,036) | |  |
|  |  |  |  |  |  |
| **Characteristic** | **N** | **(%)** | **N** | **(%)** | ***P*-value** |
| **Age (years)** |  |  |  |  |  |
| 8–12 | 638 | 49.1 | 506 | 48.8 | 0.464^a^ |
| 13–17 | 661 | 50.9 | 530 | 51.2 |  |
| **Sex** |  |  |  |  |  |
| Female | 629 | 48.4 | 528 | 51.0 | 0.119 ^a^ |
| Male | 670 | 51.6 | 508 | 49.0 |  |
| **BMI** |  |  |  |  |  |
| Underweight | 25 | 1.9 | 18 | 1.7 | 0.850 ^b^ |
| Average | 666 | 51.3 | 535 | 51.6 |  |
| Overweight | 249 | 17.5 | 193 | 18.6 |  |
| Obese | 354 | 23.2 | 290 | 28.0 |  |
| Missing/refused | 5 | 0.4 |  |  |  |
| **Race** |  |  |  |  |  |
| Non–Hispanic White | 367 | 28.3 | 306 | 29.5 | 0.810 ^b^ |
| Non–Hispanic Black | 296 | 22.8 | 220 | 21.2 |  |
| Mexican American | 303 | 23.3 | 245 | 23.6 |  |
| Other Race | 333 | 25.6 | 265 | 25.6 |  |
| **Poverty index ratio** |  |  |  |  |  |
| Below poverty level (< 1.85) | 690 | 53.1 | 606 | 58.5 | 0.754 ^b^ |
| Above poverty level (1.85-5) | 503 | 38.7 | 430 | 41.5 |  |
| Missing/refused | 106 | 8.1 |  |  |  |
| **Serum cotinine level** |  |  |  |  |  |
| < LOD | 599 | 46.1 | 394 | 38.0 | ＜0.001 ^a^ |
| ≥ LOD | 700 | 53.9 | 642 | 62.0 |  |
| **NHANES cycles** |  |  |  |  |  |
| 2013–2014 | 453 | 34.9 | 363 | 35.0 | 0.266 ^b^ |
| 2015–2016 | 491 | 37.8 | 394 | 38.0 |  |
| 2017–2018 | 355 | 27.3 | 279 | 27.0 |  |
| ^a^ Proportions were compared by Pearson chi-square test; | | | | | |
| ^b^ Mean rank was compared by Mann-Whitney U Test. | | | | | |
